# A novel technique of transpedicular opening-wedge osteotomy for treatment of rigid kyphosis in patients with ankylosing spondylitis

**DOI:** 10.1186/s12893-022-01610-2

**Published:** 2022-05-02

**Authors:** Guang Bin Zheng, Zhenghua Hong, Zhangfu Wang, Binbin Zheng

**Affiliations:** Department of Spine Surgery, Taizhou Hospital of Zhejiang Province, Wenzhou Medical University, Ximen Road 150, Linhai, 317000 Zhejiang China

**Keywords:** Transpedicular, Opening-wedge osteotomy, Ankylosing spondylitis, Thoracolumbar kyphosis

## Abstract

**Background:**

To investigate the effectiveness and feasibility of a novel vertebral osteotomy technique, transpedicular opening-wedge osteotomy (TOWO) was used to correct rigid thoracolumbar kyphotic deformities in patients with ankylosing spondylitis (AS).

**Methods:**

Eighteen AS patients underwent TOWO to correct rigid thoracolumbar kyphosis. Radiographic parameters were compared before surgery, 1 week after surgery and at the last follow-up. The SRS-22 questionnaire was given before surgery and at the last follow-up to evaluate clinical improvement. The operating time, estimated blood loss and complications were analyzed.

**Results:**

The mean operating time and estimated blood loss were 236 min and 595 ml, respectively. The mean preoperative sagittal vertical axis (SVA), thoracic kyphosis (TK), pelvic tilt (PT) and thoracolumbar kyphosis (TLK) were 158.97 mm, 51.24 mm, 43.63 mm and 41.74 mm, respectively, and decreased to 66.72 mm, 35.96 mm, 27.21 mm and 8.67 mm at the last follow-up. The mean preoperative lumbar lordosis (LL) and sacral slope (SS) were 8.30 ± 24.43 mm and 19.67 ± 9.40 mm, respectively, which increased to 38.23 mm and 28.13 mm at the last follow-up. The mean height of the anterior column of osteotomized vertebrae increased significantly from 25.17 mm preoperatively to 37.59 mm at the last follow, but the height of the middle column did not change significantly. SRS-22 scores were improved significantly at the last follow-up compared with preoperatively. Solid bone union was achieved in all patients after 12 months of follow-up, and no screw loosening, screw removal or rod breakage was noticed at the last follow-up.

**Conclusions:**

TOWO could achieve satisfactory kyphosis correction by opening the anterior column instead of vertebral body decancellation and posterior column closing, thus simplifying the osteotomy procedure and improving surgical efficacy.

**Supplementary Information:**

The online version contains supplementary material available at 10.1186/s12893-022-01610-2.

## Background

Ankylosing spondylitis (AS) is a chronic inflammatory rheumatic disease that mainly involves the axial skeleton, leading to spinal kyphosis, which decreases the patients’ quality of life [1]. Physical exercise or rehabilitation cannot prevent deformity progression; for severe spinal kyphotic patients who cannot look ahead or lie flat, surgical treatment of spinal osteotomy has been proven to be the most effective method [2–4]. Various kinds of osteotomy techniques have been introduced for spinal kyphosis correction, including Smith-Petersen osteotomy (SPO), pedicle subtraction osteotomy (PSO), closing–opening wedge osteotomy (COWO), vertebral column decancellation (VCD) and vertebral column resection (VCR) [4–8]. SPO is type of opening wedge osteotomy (OWO) that involves the use of a hinge at the posterior region of the disc space to widen the anterior disc space, which could achieve approximately 10° of correction per level. Therefore, the indication for multilevel SPO is smooth kyphosis, such as Scheuermann’s disease [5]. PSO is a type of closing wedge osteotomy (CWO) that involves shortening of the middle and posterior columns of the vertebral body; the hinge is located in the anterior aspect, and the correction angle for one level of PSO is approximately 35° [9]. PSO is the most popular technique for the treatment of rigid kyphosis, such as AS, posttraumatic kyphosis (PTK) and spinal tuberculosis. For severe and rigid kyphosis, two-level PSO showed satisfactory correction, but the surgical time and blood loss increased significantly [10]. The contact of the upper and lower surfaces of closing wedge vertebrae facilitates bone fusion for PSO, but overshortening of the posterior column can compress the dural sac and increase the risk of neurologic complications [2]. Although VCR could achieve the strongest deformity correction, the related complications were much higher than those of other techniques [11]. Zhang et al. reported a modified osteotomy technique for VCD, which was a combination of several osteotomy techniques, including SPO, PSO, COWO and eggshell techniques [4]. VCD is a Y-shaped osteotomy that shows a better kyphosis correction angle than PSO, with less decancellation of the middle column (MC) and reduced dural sac buckling [12, 13]. However, the hinge of VCD was located in the vertebral body, and the exact hinge site is not easy to control.

In this study, we introduced a new technique of transpedicular open wedge osteotomy (TOWO) for the treatment of rigid thoracolumbar kyphosis in patients with AS. The osteotomy site of TPOWO is located at the pedicle level and parallel to the vertebral endplate, the procedure of vertebral body decancellation can be abbreviated, and the hinge is located in the posterior aspect of the vertebral body. This novel osteotomy strategy simplified the surgical procedure and achieved satisfactory deformity correction. We describe the detailed steps of the TPOWO technique and evaluate the radiographic and clinical outcomes of this new technique.

## Methods

### Subjects

Eighteen AS patients (14 male, 4 female) who have thoracolumbar kyphosis and who underwent one-level TOWO from January 2011 to December 2018 at Taizhou Hospital of Zhejiang Province were reviewed retrospectively. The inclusion criteria were as follows: patients who were diagnosed with AS; global kyphosis angles that were greater than 45°; patients who showed sagittal imbalance; and follow-up of more than 12 months. The exclusion criteria were patients who were diagnosed with spinal tumor, had infections or who had a history of previous spinal surgeries; extensive aorta calcification was seen in the X-ray or CT images. This study was approved by the Institutional Review Board (IRB) of Taizhou Hospital of Zhejiang Province (K20190216).

### Radiographic measurements and clinical outcome evaluations

Freestanding whole spine and pelvis anteroposterior and lateral radiographs were obtained before surgery, one week after surgery and at the last follow-up for all patients. Three-dimensional computed tomography (3-D CT) and magnetic resonance imaging (MRI) were performed before and one week after surgery. The following radiographic parameters were measured: sagittal vertical axis (SVA: the distance between C7 plumb line and the posterosuperior corner of S1), thoracic kyphosis (TK: the Cobb angle between the superior endplate of T5 and the inferior endplate of T12), lumbar lordosis (LL: the Cobb angle between the inferior endplate of T12 and the superior endplate of S1), thoracolumbar kyphosis (TLK: the Cobb angle between the superior endplate of T10 and the inferior endplate of L2), pelvic incidence (PI: the angle between a line from the center of the femoral head to the midpoint of sacral endplate and a line orthogonal to the sacral endplate), pelvic tile (PT: the angle formed by a vertical line from the center of the femoral heads and the line through the center of the femoral heads and the midpoint of sacral endplate), sacral slope (SS: the angle between the sacral endplate and horizontal endplate), height of the anterior column of osteotomized vertebra (AC), and height of posterior column of osteotomized vertebra (PC). Sagittal translation was defined as more than 2 mm displacement of the posterior edge between the cephalad and caudal parts of the osteotomized vertebra. Bone fusion was evaluated by the standards described by Brantigan and Steffee [14]. Continuous new bone formation between the osteotomy gap in lateral X-ray or sagittal CT images was considered bone fusion. Operation time, blood loss and perioperative complication data were collected. The Scoliosis Research Society outcomes instrument-22 (SRS22) was assessed before surgery and at the last follow-up to evaluate clinical improvement.

### Surgical technique

**Fig. 1:**
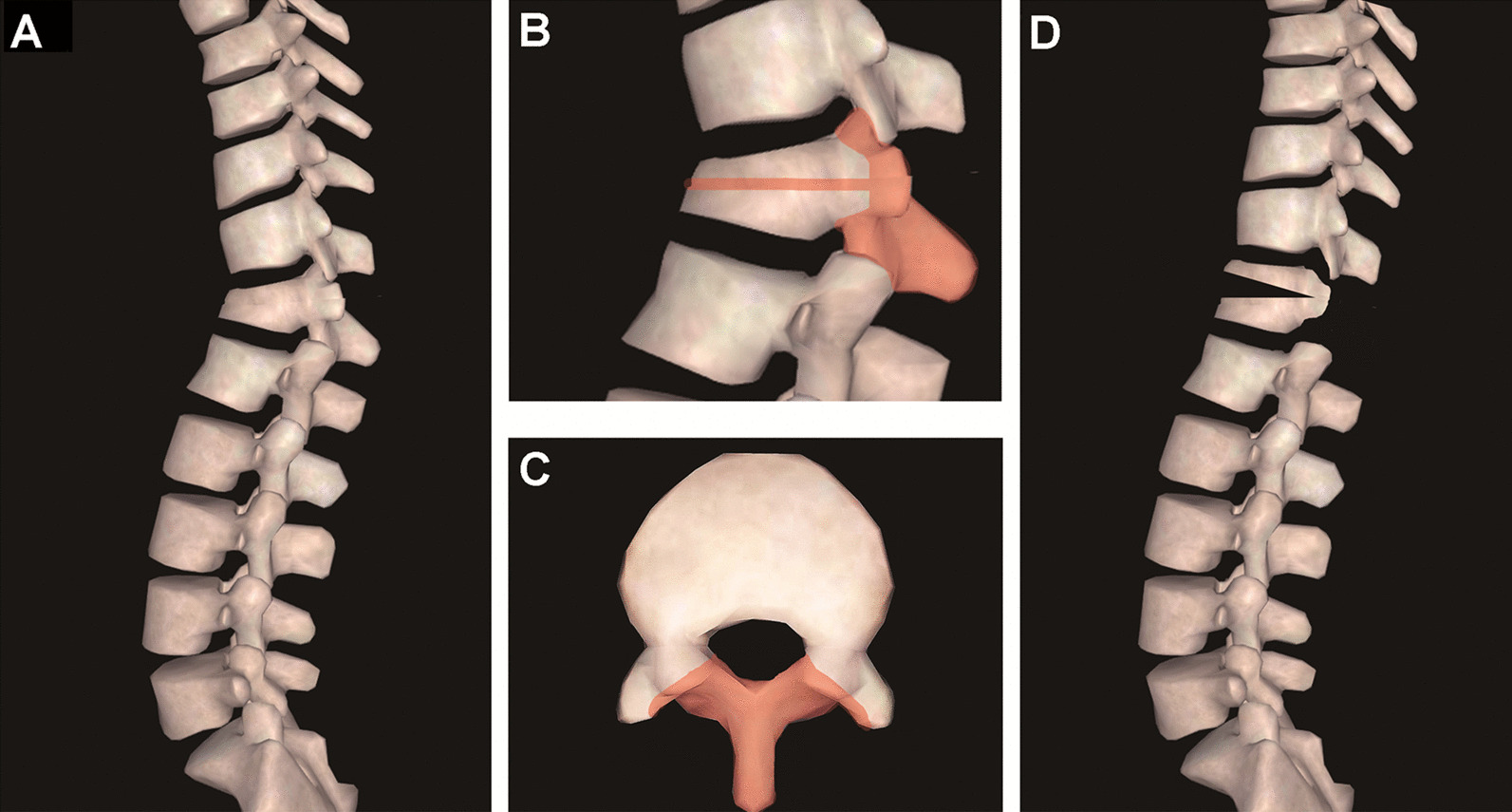
Diagrams of middle column preserved transpedicular opening-wedge osteotomy (TOWO). **a** Thoracolumbar kyphosis in sagittal vew. **b** and **c** The spinous process, lamina and facet joints were removed, and the direction of osteotomy was parallel to the endplate (the red portion). **d** The correcting hinge was located on the posterior edge of the vertebral body, which could create an open wedge of the anterior column

**Fig. 2:**
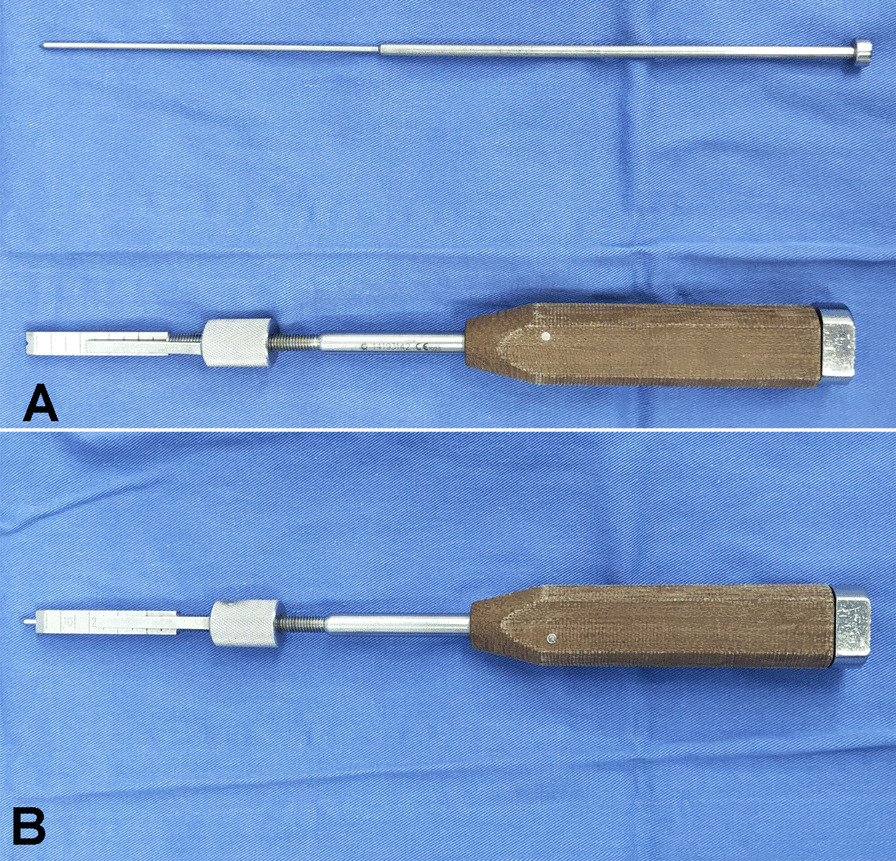
**a** The osteotome is made up of the main part with a depth-restricting device and an inner nail. The width is 8 mm, and the thickness is 4 mm. **b** After the nail is inserted into the osteotome, the top of the nail can protrude from the osteotome

**Fig. 3:**
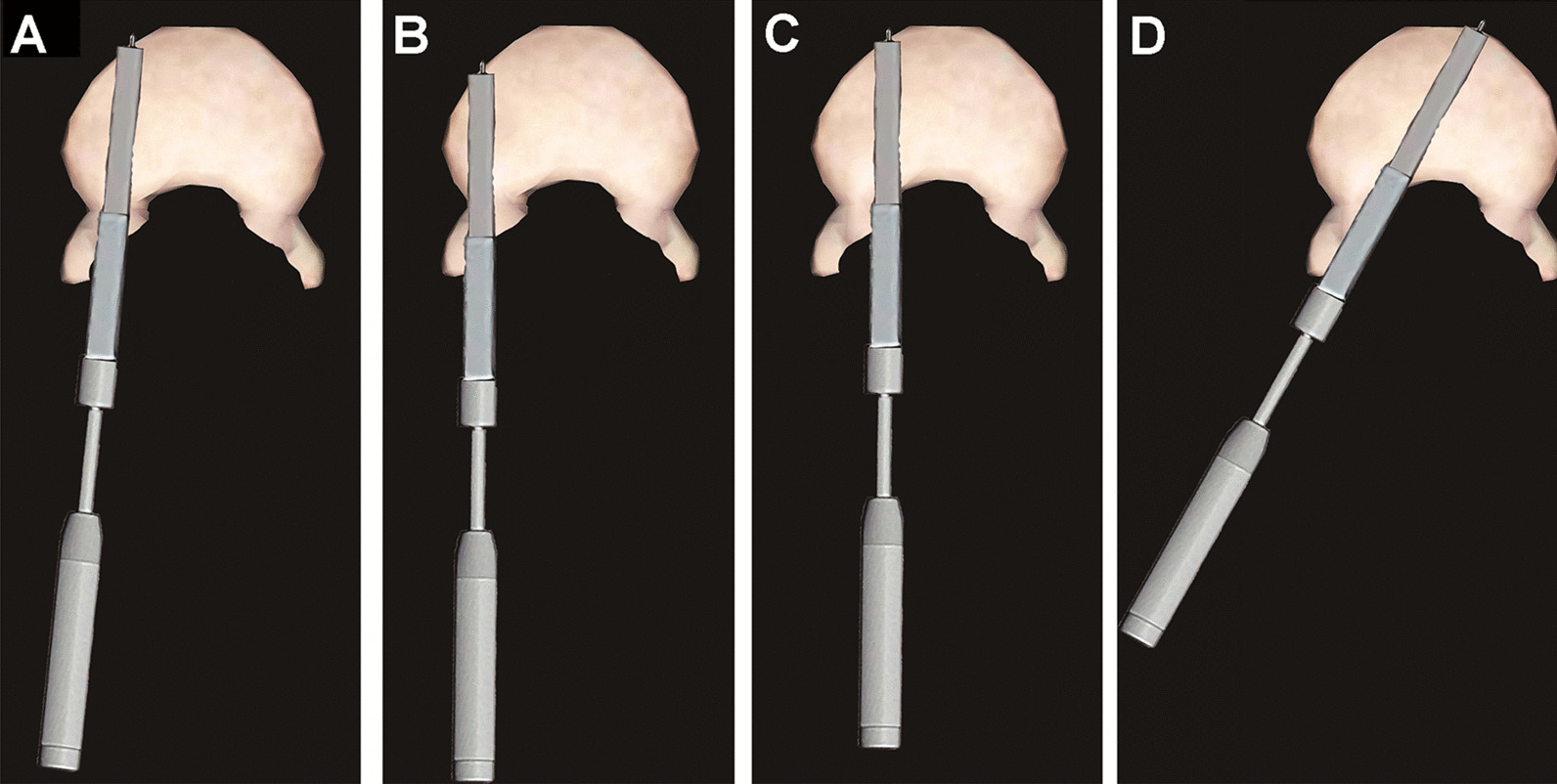
Diagrams of four-stepped osteotomy. The osteotomy was performed in four different directions to cleft the outer and inner cortex of the vertebral body on each side, and eight holes were made in the anterior cortex by projecting the nail tip to fragile the cortex. **a** The osteotome was hammered through the previous pedicle screw trajectory until the anterior edge of the osteotome touched the anterior cortex of vertebral body, an inner nail was inserted to form a hole on the anterior cortex. **b** A diverged direction of osteotomy split the outer cortex of the pedicle and vertebral body. **c** Osteotomy was performed to cleft the inner cortex of pedicle and medial part of vertebral body. **d** Osteotome was hammered at 40~45° to cleft the posterior wall of vertebral body

After general anesthesia, the patient lay prone over a reversed V-flexed operation table, and the abdomen was free by padding the chest and iliac crest to avoid vena cava compression. A standard posterior midline skin incision was made. After exposing the laminae and transverse processes to adequate levels, multiaxial long-arm pedicle screws were inserted bilaterally three segments above and three segments below the osteotomized vertebrae. Temporal pedicle screws were implanted in the osteotomized vertebrae. The screw diameter and length were estimated by measuring preoperative axial CT images to achieve maximum pull-out strength. Then, the spinous process, lamina and bilateral facet joints of osteotomized vertebrae were removed (Fig. 1). We designed an osteotome containing a mobile nail in the center of the shaft, and a depth-restricting device was installed outside the anterior part of the osteotome to restrict the osteotomy depth (Fig. 2). After removing the ipsilateral temporal pedicle screw, we measured the distance from the entry point to the anterior cortex, and the length of the anterior part of the osteotome was adjusted. The required osteotomy length could also be estimated from the preoperative CT measurement. Osteotomy parallel to the endplate was performed through the screw trajectory. A four-stepped vertebral body cleft procedure was performed for each side (Fig. 3). First, the osteotome was hammered through the previous pedicle screw trajectory until the anterior edge of the osteotome touched the anterior cortex of vertebral body, after which an inner nail was inserted to form a hole on the anterior cortex (Fig. 3A). Second, a diverged direction of osteotomy split the outer cortex of the pedicle and vertebral body, after which another hole was made on the anterior cortex by the inner nail (Fig. 3B). Third, 30–40° osteotomy was performed to cleft the inner cortex of pedicle and medial part of vertebral body, and the third hole was made on the anterior cortex by the inner nail (Fig. 3C). Fourth, the dural sac and nerve root were retracted slightly and protected by root retractor gently, the osteotome was hammered at 40–45° to cleft the posterior wall of vertebral body, with the fourth hole made on the anterior cortex (Fig. 3D). Afterward, the same procedure was repeated on the contralateral side. Neither pedicle subtraction nor vertebral decancellation was carried out. The rods were bent based on the preoperative design. After leveling the operating table smoothly, we connected the rods with the 3 cephalad pedicle screws, and the screw nuts were tightened. Subsequently, the rods were gradually pushed down to the caudal 3 pedicle screws until we heard a clear sound of cortical bone crack from the anterior cortex, which implied that the anterior cortex was open. After the rods contacted the caudal screws, the remaining nuts were tightened, and the deformity correction procedure was completed. In addition to the PSO or VCD, the hinge of TOWO was located in the posterior edge of the vertebral body (Fig. 1D). Motor- and somatosensory-evoked potential monitoring was performed routinely for all patients.

### Statistical analysis

The data were analyzed by the SPSS software package (version 23.0, IBM Corp., Chicago, IL, USA). One-way ANOVA was used to evaluate the preoperative, postoperative and last follow-up radiographic parameters. For nonparametric data, Kruskal–Wallis tests were used. Preoperative and last follow-up SRS-22 scores were evaluated by paired t tests. P < 0.05 was considered statistically significant.

## Results

### Surgical outcomes

**Fig. 4:**
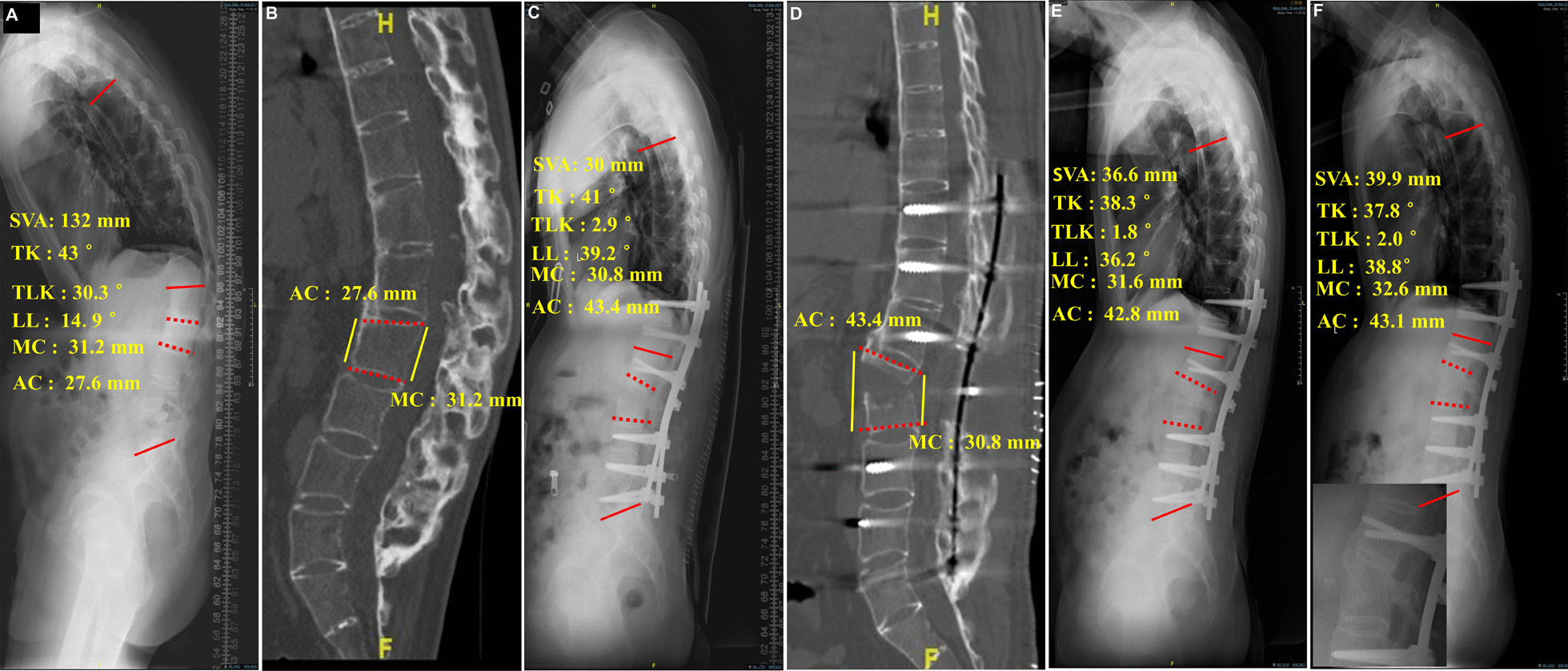
A 36-year-old male patient with thoracolumbar kyphosis secondary to ankylosing spondylitis. **a** and **b** Thoracic kyphosis (TK), lumbar lordosis (LL), thoracolumbar kyphosis (TLK), sagittal vertical axis (SVA), height of the middle column (MC) and anterior column (AC) were 43°, 14.9°, 30.3°, 132 mm, 31.2 mm and 27.6 mm, respectively, before surgery. **c** and **d** One week after surgery, the TK, TLK and SVA decreased to 41°, 2.9° and 30 mm, respectively, the LL and height of AC increased to 39.2° and 43.4 mm, respectively, and the height of MC was 30.8 mm, which was similar to the preoperative value. **e** and **f** At the 4-month and 2-year follow-ups, the improved values were adequately maintained, and the heights of the AC were 43.1 mm and 42.6 mm, respectively; at the 2-year follow-up, new bone formation could be observed between the gaps of open wedged vertebrae, which implied that bone union was achieved

All patients received one-level osteotomy, and the osteotomy level was T10 for 1 case, T12 for 3 cases, L1 for 7 cases, L2 for 5 cases and L3 for 2 cases. The mean operating time and estimated blood loss were 236 min (range 185–320 min) and 595 ml (range 400–900 ml), respectively. The mean follow-up time was 33 months (range of 24–49 months). No intraoperative acute complications such as massive bleeding or great vessel injury occurred. Three patients experienced cerebrospinal fluid (CSF) leakage because of the adhesion between the dura mater and ligament flavum during laminectomy. After bed rest and conservative treatment, all the patients recovered within 2 weeks. One patient showed lower extremity numbness, but the symptoms disappeared after 2 months. No deep wound infection or paralysis occurred in any patients. Solid fusions were achieved in all the patients after 12 months according to radiographic evaluation. The gaps between osteotomized vertebrae were filled with newly formed bone (Fig. 4F). No screw loosening or screw or rod breakage was noticed during the follow-up (Additional file 1: Video S1).

### Radiographic and clinical results

The preoperative SVA, TK, PT and TLK values were 158.97 ± 55.80 mm, 51.24 ± 18.21 mm, 43.63 ± 5.30 mm and 41.74 ± 11.16 mm, respectively; one week after the operation, these parameters decreased to 63.02 ± 37.58 mm (P < 0.001), 33.02 ± 11.45 mm (P < 0.001), 25.91 ± 8.48 mm (P < 0.001) and 7.37 ± 7.46 mm (P < 0.001), respectively, and were adequately maintained until the last follow-up. The preoperative LL, SS and height of AC of osteotomized vertebrae were 8.30 ± 24.43 mm, 19.67 ± 9.40 mm and 25.17 ± 3.94 mm, respectively; however, at one week after the operation, these parameters increased to 36.61 ± 13.73 mm (P < 0.001), 29.12 ± 8.82 mm (P < 0.001) and 39.51 ± 5.65 mm (P < 0.001), respectively, and were maintained adequately at the last follow-up. However, the height of the MC of osteotomized vertebrae did not change significantly preoperatively, at one week after surgery or at the last follow-up (P = 0.507) (Table 1) (Fig. 4). The SRS-22 questionnaire scores improved significantly at the last follow-up compared with before the operation (Table 2).

**Table 1:** Radiographic assessment of preoperative, postoperative and last follow-up data

Parameters	Preoperative	Postoperative	Last follow-up	*P*
SVA (mm)	158.97 ± 55.80	63.02 ± 37.58^a^	66.72 ± 47.84^b^	< 0.001
LL (°)	8.30 ± 24.43	36.61 ± 13.73^a^	38.23 ± 15.66^b^	< 0.001
TK (°)	51.24 ± 18.21	33.02 ± 11.45^a^	35.96 ± 11.35^b^	< 0.001
PT (°)	43.63 ± 5.30	25.91 ± 8.48^a^	27.21 ± 8.22^b^	< 0.001
SS (°)	19.67 ± 9.40	29.12 ± 8.82^a^	28.13 ± 6.79^b^	< 0.001
TLK (°)	41.74 ± 11.16	7.37 ± 7.46^a^	8.67 ± 7.35^b^	< 0.001
AC (mm)	25.17 ± 3.94	39.51 ± 5.65^a^	37.59 ± 6.25^b^	< 0.001
MC (mm)	28.08 ± 3.20	27.22 ± 4.01	26.41 ± 4.24^b^	0.507

*SVA* sagittal vertical axis, *LL* lumbar lordosis, *TK* thoracic kyphosis, *PT* pelvic tilt, *SS* sacral slope, *TLK* thoracolumbar kyphosis, *AC* anterior column, *MC* middle column

^a^Significant difference between postoperative and preoperative: p < 0.001

^b^Significant difference between last follow-up and preoperative: p < 0.001

**Table 2:** Comparison of preoperative and last follow-up data of Scoliosis Research Society-22 (SRS-22) outcomes

Parameters	Pain	Function	Appearance	Mental	Satisfaction
Pre-operative	1.71 ± 0.40	2.27 ± 0.43	1.94 ± 0.58	1.79 ± 0.37	1.53 ± 0.30
Last follow-up	4.27 ± 0.31	4.34 ± 0.35	4.32 ± 0.42	4.39 ± 0.33	4.64 ± 0.33
*p*	< 0.001	< 0.001	< 0.001	< 0.001	< 0.001

## Discussion

The highlight of TOWO is that the osteotomy procedure is simplified by two major steps: transpedicular transection of the vertebral body with osteotomy and correction of kyphosis by the hinge located in the posterior aspect of the vertebral body. To improve the surgical efficiency, we utilized a special osteotome that contained a nail in its center. When the anterior edge of the osteotome reached the anterior cortex of the vertebral body, we hammered the inner nail to create holes in the anterior cortex to make it fragile. Only 4 steps were needed to complete the osteotomy procedure on each side by changing the directions of the osteotome. Therefore, curettage of intervertebral cancellous bone is unnecessary, and the whole osteotomy procedure takes only 10–20 min. The radiographic results indicated that single-level TOWO showed more than 40° of sagittal deformity correction on average. It has been reported that the maximum deformity correction angle for one-level PSO should be less than 40°; if overcorrection was attempted, acute shortening of the dural sac would affect the vascular circulation of the spinal cord [2]. Furthermore, overcompression of the dural sac could induce neurologic complications [15]. Kawahara et al. performed an experiment using dogs to clarify the effects of acute thoracolumbar (T13) shortening on spinal cord evoked potential and spinal cord blood flow [16]. The results showed that the dural sac shrank and buckled after one-third to two-thirds of vertebral column shortening, and more than two-thirds of vertebral column shortening increased the potential for neurologic deficits and decreased the blood flow of the spinal cord. A recent study of the middle thoracic shortening test in a canine model also showed that the safe range was within 1/3 of a vertebral height shortening with two-segment laminectomy [17]. Accordingly, for severe kyphosis patients who require a correction angle greater than 40°, single-level PSO cannot achieve satisfactory kyphosis correction; however, two-level PSO could provide enough correction angle, but the blood loss and operation time increased significantly [10]. To overcome these shortcomings, modified osteotomy techniques were investigated. Wang et al. presented a new VCD technique with the hinge located in the vertebral body [4, 13, 18, 19]. Compared with PSO, VCD could provide a 10° larger correction angle with 10 mm less shortening of the MC. VCD is an alternative to PSO for correcting severe kyphosis, but MC shortening still cannot be avoided completely, and accurate decancellation of the vertebral body is not easy to control.

Compared to the abovementioned osteotomy techniques, our technique could minimally avoid posterior column shortening. No neurologic deficits were observed during or after surgery in this study. Although this is a retrospective study without a control group, the blood loss and operation time were significantly lower than those in previous studies of PSO or VCR [8, 19]. The preservation of cancellous bone and close contact between the posterior edge of osteotomized vertebrae may decrease blood loss from the cancellous bone. Additionally, we did not dissect the paravertebral mussels around the osteotomized vertebra. The integrity of muscles and soft tissues decreased the translation of the osteotomized surface during the osteotomy and subsequent mechanical correction [20]. The preservation of cancellous bone of MCs could also increase spinal stability and benefit bone union between osteotomized surfaces. In this study, none of the patients showed sagittal or lateral translation of the osteotomized site during surgery or the follow-up period, and the radiographic study after 12 months showed that sufficient new bone formation was observed between the osteotomized gaps in all patients. At the last follow-up, the correction was maintained, and no instrumentation failure, including rod or pedicle screw breakage, was observed. These results indicate that TOWO might be a safe and effective technique for correcting thoracolumbar kyphosis in ankylosing spondylitis.

Aortic injury should be highly considered in OWO compared to CWO [21]. Aortic injury is a catastrophic complication in osteotomy surgery. It may be correlated with the elongation of the aorta during deformity correction or the translation of the osteotomized surfaces, which is not stabilized by internal fixation, especially when the elasticity of the arterial wall is reduced by atheromatous deposits or calcification [22]. Weatherley et al. reported two cases of vascular injury associated with closing wedge osteotomy for AS in 1988 [23]. Both of them suffered aorta rupture, which was confirmed in the postmortem findings. The other three cases of aortic rupture after spinal osteotomy were reported in 1956, 1971 and 1986 [24–26]. Overdistraction of the aorta during surgery was considered to be the possible mechanism of aorta rupture. However, recent studies showed that although the length of the aorta increased more than 2 cm after osteotomy, no aorta rupture occurred when internal fixation was applied [27, 28]. Liu et al. reported that the length of the aorta was increased approximately 2 cm for 39° of kyphosis correction in closing wedge osteotomy [28]. Chang et al. also reported that the mean aorta lengthening increased to 2.8 cm (range or 1.7–3.5 cm) at COWO [6]. In our study, the mean increase in the height of the anterior column was 1.4 cm on average, and no aorta rupture occurred during or after surgery in our patients. We hypothesized that translational shear force during deformity correction might be a higher risk factor than vessel lengthening for aortic injury. In Weatherley’s report of two patients with aortic injury, internal fixation was not added for one patient, and Harrington instrumentation was applied for another patient. Without strong internal segmental instrumentation, translational movement of the osteotomized surface still exists. However, the exact mechanism of aortic injury during spinal kyphosis correction needs further experimental support. Although pedicle screws and temporary rods could provide sufficient stability to the osteotomized interface, the correction procedure should be performed smoothly to avoid repeated irritation or sudden distraction of the aorta. To improve safety, we created holes in the anterior cortex of the vertebral body using our specially designed osteotome. These holes made the anterior cortex fragile and vulnerable to bending and distraction forces, which avoided the sudden splitting of the vertebral body and sharp bone spur formation. Although great vessel-related complications caused by spinal osteotomy are rare, the potential risk of aortic injury should be highly considered in patients who undergo open wedge osteotomy. This study has some limitations, as it is a retrospective study with a small number of cases, and no control group was included. Therefore, a controlled prospective study is needed to further prove the effectiveness, safety and long-term outcomes of this technique.

## Conclusion

Transpedicular opening-wedge osteotomy (TOWO) might be a safe and effective strategy for correcting rigid thoracolumbar kyphosis secondary to AS. MC preservation minimizes the shortening of the posterior column and prevents overcompression of the dural sac, which also reduces operating time and decreases blood loss. This modified and simplified osteotomy technique is easy to master and popularize.

## Supplementary Information

**Additional file 1.** Video S1.

## Data Availability

The datasets used and analysed during the current study available from the corresponding author on reasonable request.

## Abbreviations

TOWO: Transpedicular opening-wedge osteotomy
AS: Ankylosing spondylitis
SVA: Sagittal vertical axis
TK: Thoracic kyphosis
LL: Lumbar lordosis
TLK: Thoracolumbar kyphosis
PT: Pelvic tilt
SS: Sacral slope
AC: Anterior column
MC: Middle column
SRS: Scoliosis research society
SPO: Smith-Petersen osteotomy
PSO: Pedicle subtraction osteotomy
VCR: Vertebral column resection
VCD: Vertebral column decancellation
OWO: Opening wedge osteotomy
CWO: Closing wedge osteotomy
